# Planning, construction and use of handmade simulators to enhance the teaching and learning in Obstetrics*

**DOI:** 10.1590/1518-8345.3684.3302

**Published:** 2020-07-01

**Authors:** Roxana Knobel, Mariane de Oliveira Menezes, Débora de Souza Santos, Maíra Libertad Soligo Takemoto

**Affiliations:** 1 Universidade Federal de Santa Catarina, Departamento de Ginecologia e Obstetrícia, Florianópolis, SC, Brazil.; 2 Universidade Estadual Paulista, Faculdade de Medicina, Botucatu, SP, Brazil.; 3 Scholarship holder at the Coordenação de Aperfeiçoamento de Pessoal de Nível Superior (CAPES), Brazil.; 4 Universidade Estadual de Campinas, Faculdade de Enfermagem, Campinas, SP, Brazil.; 5 Universidade Estadual Paulista, Departamento de Enfermagem, Botucatu, SP, Brazil.

**Keywords:** Simulation, Obstetrics, Teaching, Teaching Materials, Education, Nursing, Training, Simulação, Obstetrícia, Ensino, Materiais de Ensino, Educação em Enfermagem, Capacitação, Simulación, Obstetricia, Enseñanza, Materiales de Enseñanza, Educación en Enfermería, Capacitación

## Abstract

to describe the development process and present the results of a pilot study on the use of low-cost handmade simulators for teaching and learning Obstetrics.

**Method:**

presentation of 3 low-cost simulators designing, based on educational needs identified in real-world training contexts. The developing process is presented in detail and each simulator was tested and re-tested, being submitted to improvements until their final version. The simulators presented are: delivery simulator shorts, Neoprene uterus for postpartum hemorrhage management, and perineal repair simulator. A pilot study was carried out to evaluate the perception of apprentices through a structured questionnaire, using the Kirkpatrick evaluation model. Data were descriptively analyzed.

**Results:**

the respondents (31 apprentices) positively evaluated the simulators, perceiving significant gains in theoretical knowledge, ability to solve clinical problems and decreased anxiety to deal with situations similar to those simulated.

**Conclusion:**

low-cost, handmade simulators are feasible and effective, resulting in positive learner evaluations. Their availability as open technology allows the dissemination of their use.

## Objetivo

descrever o processo de desenvolvimento e apresentar os resultados de um estudo piloto sobre o uso de simuladores artesanais de baixo custo para ensino e aprendizagem de obstetrícia.

**Método:**

apresentação do processo de desenvolvimento de três simuladores de baixo custo, a partir de necessidades educacionais identificadas em contextos reais de formação. O processo de elaboração é apresentado em detalhes e cada simulador foi testado e re-testado, sendo submetido a processos de melhorias até sua versão final. Os simuladores apresentados são: bermuda simuladora de parto, útero de Neoprene para tratamento de hemorragias e simulador de sutura de lacerações perineais. Foi realizado estudo piloto de avaliação da percepção dos aprendizes, através de questionário estruturado, empregando o modelo de avaliação de Kirkpatrik. Os dados foram analisados de forma descritiva.

**Resultados:**

os respondentes (31 aprendizes) avaliaram positivamente os simuladores, tendo percebido ganhos significativos em conhecimento teórico, habilidade para resolver problemas clínicos e diminuição da ansiedade para lidar com situações semelhantes às simuladas.

**Conclusão:**

simuladores artesanais e de baixo custo são factíveis e efetivos, resultando em avaliações positivas dos aprendizes. A disponibilidade dos mesmos como tecnologia aberta permite a disseminação de seu uso.

describir el proceso de desarrollo y presentar los resultados de un estudio piloto sobre el uso de simuladores artesanales de bajo costo para la enseñanza y el aprendizaje de la obstetricia.

**Método:**

presentación del proceso de desarrollo de tres simuladores de bajo costo, basado en necesidades educativas identificadas en contextos de capacitación reales. El proceso de elaboración se presenta en detalle. Cada simulador fue testeado una y otra vez y se sometió a procesos de mejora hasta su versión final. Los simuladores presentados son: pantalones cortos simuladores de parto, útero de neoprene para tratamiento de sangrado y simulador de sutura de laceraciones perineales. Se realizó un estudio piloto para evaluar la percepción de los alumnos, utilizando un cuestionario estructurado y se siguió el modelo de evaluación Kirkpatrik. Se practicó el análisis descriptivo de datos.

**Resultados:**

los encuestados (31 aprendices) evaluaron positivamente los simuladores, habiendo notado ganancias significativas en el conocimiento teórico, capacidad para resolver problemas clínicos y disminución de la ansiedad para lidiar con situaciones similares a las simuladas.

**Conclusión:**

los simuladores artesanales de bajo costo son factibles y efectivos, lo que resulta en evaluaciones positivas de los aprendices. Su disponibilidad como tecnología abierta permite la difusión de su uso.

## Introduction

Implementation of good obstetric practices and prevention of maternal and newborn morbidity and mortality are the focus of obstetric care. Thus training and education of health care providers towards better intrapartum practices and effective emergency management are key-points to improve care^(^ 1 ^)^. In this context, research and experimentation aimed at teaching good obstetric practices are particularly relevant for Nursing, considering the historical and growing insertion of direct-entry midwives and nurse midwives (MNM) in maternal and neonatal care in Brazil and worldwide^(^ 2 ^)^.

Since the 1970s, the Pan American Health Organization and the World Health Organization (PAHO/WHO) have described the roles and activities of MNM, which cover direct care, management, education and research. A report examining the current state of Midwifery and Obstetrics in 73 low- and middle-income countries, including Brazil, presented in 2014 by the United Nations Population Fund (UNFPA) in collaboration with the WHO and the International Confederation of Midwives (ICM), reports that urgent investment is needed to improve the quality of obstetric care to prevent about two-thirds of all maternal and newborn deaths - which would save millions of lives each year. The countries selected for the report (African, Asian and Latin American) are responsible for 96% of all global maternal deaths, 91% of fetal deaths and 93% of newborn deaths. The goals include adequate access for women to midwifery services; high-quality primary care and the possibility of transfer to next level when needed; increased availability of MNM and its beneficial interventions for the mother and baby dyad; and strengthening MNM local associations^(^ 3 ^)^.

Given the growing recognition of MNM role in the implementation of good obstetric practices, aiming to improve quality of care and prevent health problems with relevance from the public health perspective, new challenges associated with training and continuing education of these Nursing providers are highlighted. Therefore, studies addressing simulation-based Nursing education demonstrate its potential in enabling the student to deal with situations of anxiety and stress typical of nursing clinical practice. Besides aspects of theoretical knowledge, technical skills and critical thinking, students may experience emotional, spiritual and ethical issues regarding the care for patients and their families, involved in the context of nursing simulation^(^ 4 ^)^.

In Nursing teaching, simulation-based educational practices have been reinforced in the national and international literature, as it allows an ethically appropriate approach and promotes patient safety, considering that the first experience of care will not be carried out with a real patient^(^ 5 ^)^. When teaching undergraduate students, not all interventions can be performed autonomously by them (such as emergency situations), so the simulation educational activities provide the opportunity to experience events that would not be possible in real-world settings^(^ 4 ^-^ 6 ^)^. Simulation environments replicate a controlled clinical scenario allowing detailed observation of students in action, feedback and repetition as many times as needed without any harm to patients^(^ 5 ^)^.

The use of simulators and simulation environments for teaching health professionals is well established in the literature^(^ 5 ^,^ 7 ^-^ 8 ^)^. Although the quality of studies is heterogeneous and with several different indicators, evidence has shown that simulation-based teaching is effective and leads to better and more lasting results than traditional teaching^(^ 7 ^,^ 9 ^)^. Its use can improve clinical, technical, communication, and teamwork skills, improve performance and reduce errors^(^ 5 ^)^. There is evidence that simulation-based medical education can improve both learning and patient care, as well as the clinical practice and still have a positive effect on public health^(^ 8 ^,^ 10 ^)^.

Particularly in Obstetrics teaching, the use of simulators and simulation environments has been studied in various settings^(^ 8 ^)^, mostly obstetric emergencies such as shoulder dystocia^(^ 11 ^-^ 12 ^)^, postpartum hemorrhage^(^ 13 ^-^ 18 ^)^, pre-eclampsia and eclampsia^(^ 16 ^-^ 17 ^)^. There is also evidence on simulation to enhance surgical skills such as suturing of vaginal and severe lacerations^(^ 19 ^)^. A 2014 literature review showed that after simulations, it was possible to observe an increase in knowledge as well as technical, communication and teamwork skills^(^ 16 ^)^. A 10-year follow-up study of a simulation-based training for shoulder dystocia management identified an increasing in the number of diagnosis and a decreasing in the number of neonatal brachial plexus lesions^(^ 11 ^)^. At least one study conducted in Mexico^(^ 1 ^)^ was able to demonstrate that the implementation of a continuing education program including simulations has successfully modified obstetric practice with better results in terms of good practices adoption. Another training program with simulation in Tanzania showed a 47% reduction in postpartum blood transfusion rates^(^ 13 ^)^.

Simulation environments and high-fidelity simulators, although proven to be useful, find barriers to their effective use in teaching, the main one being their cost^(^ 5 ^,^ 17 ^)^. With well-established learning objectives, the use of low-cost, handmade simulators can be a viable and effective alternative in the teaching and learning process^(^ 15 ^,^ 17 ^-^ 21 ^)^. Thus, there are several examples of simulators handmade produced and at a reduced cost, both for teaching obstetrics^(^ 17 ^-^ 19 ^,^ 22 ^)^ and other specialties/situations^(^ 23 ^)^. There is no evidence demonstrating that the hyper-reality of the simulator improves participant learning, the low cost and sometimes even the low fidelity of a simulator does not seem to represent an obstacle to its use^(^ 20 ^-^ 21 ^)^.

However, cultural differences in the curriculum of Nursing schools do not allow us to state with the existing data if the use of handmade simulators is effective for teaching and learning, requiring further study. Additionally, studies in the Brazilian setting addressing low-cost simulators in the context of Obstetrics teaching were not found.

Considering this broader context, the objectives of this article are: to describe the creation and use of handmade simulators for teaching Obstetrics and to present the results of a pilot study on the use of handmade simulators as educational technologies, through the perceptions of nursing professionals (MNM), obstetrics resident physicians and undergraduate students (apprentices) who participated in classes using the simulators.

## Method

This is a cross-sectional pilot study to assess apprentices’ perception about the use of low-cost handmade simulators for Obstetrics teaching, based on the hands-on teaching experience of the developers. The scope of the analysis is characterized as a study to improve the quality of health care, through the improvement of teaching and learning of health professionals. Thus, the SQUIRE guidelines for publications of this type were adopted. The methods section will be discussed in two stages: the process of developing the simulators and the methods used for the pilot study.

The simulators were created or adapted (based on ideas suggested through panels of experts and/or available on the Internet) and made by the authors, based on educational needs identified in hands-on training workshops for professionals and students of Midwifery and Obstetrics offered systematically since 2014. All simulators have a Creative Commons license and are available to be reproduced or adapted in other services on the website http://saudesimuladores.paginas.ufsc.br/.

The simulators presented in this article are: (i) Birth simulator shorts with a doll; (ii) Neoprene uterus simulator for the management of postpartum hemorrhage; (iii) Simulator of perineal tear repair.

The process of setting the simulators began with the identification of an educational need observed in a real-world training context. From this need, there was a seek for already existing alternatives, through the search of previous simulators. The search sources were films and websites with content on Obstetrics and Obstetrics training, in addition to the constant exchange of ideas and suggestions from peers. Based on this search, the feasibility of a simulator was evaluated and, when it seemed feasible - in terms of cost, the possibility of material acquisition and maintenance, size -, a prototype was developed and tested. The tests were made through expert evaluations (professionals of notorious knowledge in the area of Obstetrics and who work in the hands-on training of other professionals). The aim was to establish face (to evaluate the realism, aspect) and content (pedagogical value, effectiveness in solving the proposed problem) validity^(^ 5 ^,^ 24 ^)^. It should be noted that the face and content validity depends on the learning objective. A piece of curvy fabric (synthetic leather), for example, does not have the appearance of human skin, but with the proper assembly may be valid for suturing training. The structure of the simulation is considered to be a process, not a product, and the simulation training needs strategic planning in order to be effective^(^ 14 ^)^.

On many occasions, tests have determined changes in the prototype. This was redone and retested as many times as was necessary. At the time we obtained a version of each simulator that was considered appropriate, they were considered finished and available for implementation. Even after implementation, it must be considered that the simulators should continue to be tested and may undergo improvements and modifications. We hope that the simulators are recreated and adapted to different realities, with citation of the original authorship.

**Figure 1:**
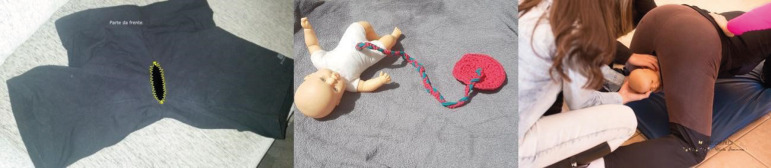
Birth simulator shorts with a doll (short with sewing scheme, baby doll with crochet placenta and shoulder dystocia simulation)

For the birth simulator shorts with a doll, the educational need identified was the need for training maneuvers that require changing the woman’s position, such as for shoulder dystocia^(^ 25 ^)^ and emergency breech vaginal birth^(^ 26 ^)^. The rigid plastic or rubber birth simulators (maternal pelvis and fetus) do not allow these maneuvers to be performed adequately and make it difficult to train the provider/birthing woman interaction, including the dynamic effects of the birthing woman movements on dystocia management. The model consists of a *Lycra* shorts with a hole and zigzag stitching that will simulate the maternal perineum at birth. The doll used is a toy baby bought in children’s toy stores (hard plastic limbs and head and cloth body, size to a newborn) with a placenta made of crochet, as shown in Figure 1.

One student or actress wears the shorts and may experience the birth, while another student may attend the birth, identify and manage dystocias. This simulator allows the training of skills and attitudes related to normal delivery care, resolution of dystocia and obstetric complications (particularly useful for dystocia requiring a maternal change of position). It was also used for the discussion of models of care and communication skills, as it allows apprentices to play the birthing person role. It is a low-cost solution with a final cost of approximately USD 26.91 (conversion rate of 1 USD = BRL 3.97, as of May 29, 2019) and allows the presentation and simulation in various environments - schools, pregnant women groups, and professional training environments.

Regarding the Neoprene uterus simulator, the identified educational need was skills and attitudes training for the management of postpartum hemorrhage, which is a high prevalence condition and one of the leading causes of maternal deaths in Brazil^(^ 27 ^)^. There are simulators on the market, even tested with excellent results^(^ 13 ^,^ 28 ^)^. However, although they are considered low-cost (compared to others in the market), they are not affordable in our reality.

**Figure 2:**
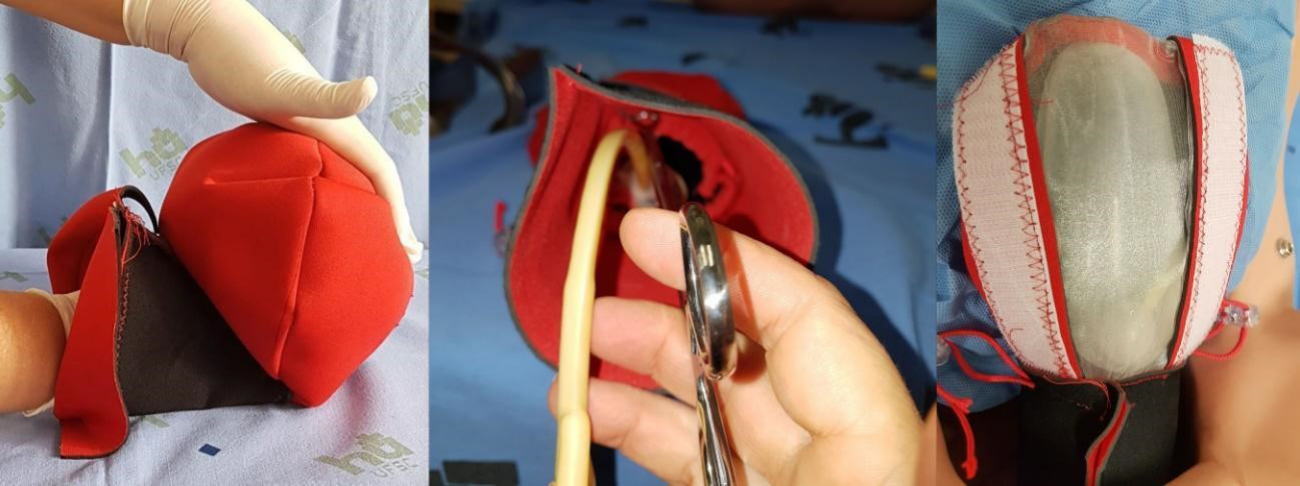
Neoprene uterus simulator for postpartum hemorrhage treatment in bi-manual compression; uterine tamponade balloon insertion; balloon visualization

A uterus simulator made of Neoprene fabric (synthetic polychloroprene elastomer) was created in a domestic sewing machine. Initially, a Neoprene model was made with a semi-inflated plastic ball insert that allows simulating the uterine contractility when squeezed. This model can be used to simulate the diagnosis of uterine hypotonia, performing abdominal uterine massage, bimanual uterine compression (concomitant vaginal and abdominal pressure)^(^ 27 ^)^. Also, when the instructor’s hand compresses the ball internally, the return of uterine tonus after treatment is also simulated. After the initial tests, the same model presented in Figure 2 was improved to allow the insertion of a uterine tamponade balloon, which is an important conservative measure for postpartum hemorrhage treatment, as recommended in national and international guidelines^(^ 27 ^)^.

The production cost is approximately USD 17.63 *per* unit. This simulator was validated by 18 specialists in the field (doctors and midwives) who considered that it replicates the anatomical structures and tactile sensation of the abdominal and bi-manual uterine compression^(^ 29 ^)^.

The simulator can be used on a workbench to visualize/feel/train the technique of massage and balloon insertion. It also fits to be placed in shorts similar to the birth simulator shorts - adapted with Velcro - for the simulation of postpartum hemorrhage with an actress as the patient, which allows training of the various management steps, communication with the patient and teamwork. This simulation performed in a real environment (labor and delivery room) allows for recognizing difficulties (of material access, communication, task division) and search for solutions.

Perineal tears can occur during vaginal births, usually spontaneous lacerations with varying degrees of severity, most of which are superficial without need for treatment. Some second-degree tears (or less extensive lacerations with anatomical issues or active bleeding) require suturing, which can be performed by both nursing professionals and doctors. The best technique for suturing second-degree tears (involving skin and/or mucosa and muscles) is continuous suturing in all planes with a single suture thread^(^ 30 ^)^ and many professionals are not familiar with this technique.

Severe tears (third and fourth degree) reach the external anal sphincter and rectal mucosa, respectively. They are less frequent and have a potential for permanent sequelae and pathologies if not properly repaired^(^ 19 ^)^. The obstetrician is technically responsible for this repair, but many residents and trained professionals feel insecure and unable to perform the suturing^(^ 19 ^)^, due to its rare need and their little exposure to the procedure during training.

**Figure 3:**
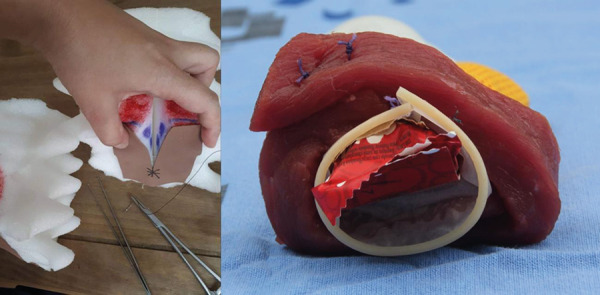
Second-degree perineal laceration and severe perineal laceration simulator

Thus, regarding the suturing simulator of second-degree and severe perineal tears, the educational need found was therefore training for postpartum perineal repair. Several models were tested and two different simulators were proposed. A foam model for training second-degree perineal lacerations and a model with a male condom, bovine tissue, and beef for 3^rd^ and 4^th^-degree lacerations, as shown in Figure 3. These simulators do not allow simulating the clinical situation but allow training specific knowledge and skills of suturing techniques. The foam simulator has a cost of approximately USD 5.03 and the severe laceration costs approximately USD 2.52.

To evaluate the simulators, three different groups of learners completed a structured questionnaire: undergraduate medical students, gynecology and obstetrics resident doctors, and nurse midwives, who attended obstetric emergencies training using the simulators. The only inclusion criterion for answering the questionnaire was having participated in a class with simulation, using at least one of the simulators. The simulators have been developed and employed in hands-on simulation workshops since 2014 and the study was conducted in March 2019.

Participants were invited to participate voluntarily and without any benefits or harms to their activities. All volunteers were informed about the study aims and signed the Informed Consent Form. A semi-structured questionnaire was created by the authors specifically to evaluate the simulators. It was anonymously answered by the respondents themselves and contained questions to assess the reaction, self-perception of learning and behavior, in addition to questioning about the immersion in the clinical situation that the simulator allowed. It consisted of eight closed-ended questions using a *Likert* scale. The questionnaire also presented an open-ended question on the use of acquired knowledge, skills, and attitudes in real-world situations and an open space for observations.

There are several models for the evaluation of the simulation process effectiveness, and Kirkpatrick’s model is one of the most used. This model, initially proposed by the author in the 1960s, is widely accepted and used since then^(^ 8 ^,^ 31 ^)^. The popularity of this model is due to the fact that it results in a well-defined system for demonstrating the results of an intervention or training^(^ 31 ^)^. Although it is subject to criticism (may be incomplete/may assume unrealistic causal associations), it simplifies the complex process of training evaluation^(^ 32 ^)^. To measure the impact of the intervention, questions were formulated based on the Kirkpatrick’s assessment model, which proposes 4 levels of assessment: reaction (evaluates participants’ reaction to the training program), learning (knowledge, skills, and attitudes acquired through the training), behavior (which knowledge and/or skills acquired through the training were applied to the learner’s work and/or resulted in improved performance) and outcomes (impact of the training on the resolution of an existing problem and/or organizational indicators/objectives)^(^ 8 ^-^ 31 ^)^. The closed-ended questions sought to evaluate the reaction (satisfaction and immersion of the learner in the simulation), the learning (theoretical, practical and general), behavior (improvement in skills, decrease in stress) and the outcomes (ability to solve clinical situations after the simulation). The open-ended question sought to assess at the qualitative level whether and how the learners used the learnings obtained from the simulations in real situations, to analyze both behavior and outcomes levels of Kirkpatrick’s model.

The quantitative data were transcribed to a spreadsheet and analyzed using descriptive statistics. Since this is a pilot study, no hypothesis testing was employed and sample size calculation is not applicable.

The qualitative analysis of the open-ended question had a complementary role to the study and was guided by the content analysis method^(^ 33 ^)^, using the thematic modality to reveal the meaning of the experience for the participants. We developed the stages of pre-analysis, material exploration, treatment of the results and interpretation, and then proceeded: 1) data ordering, which included transcription, rereading and organization of the answers; 2) categorization of the information, after exhaustive and repeated readings of the texts, to define the category of analysis; and 3) The third stage was developed through the observed relationship between the interpretation of answers and the evaluation issued by the participant, which allowed the design of the following category of analysis: “The training helps the care provider to feel safer and more confident”.

Ethical requirements applicable to research involving human beings were respected, and the project was approved under the record number 71272017.4.0000.0121 of the Ethics Committee of the Federal University of Santa Catarina, in compliance with Resolution 466/2012 of the National Health Council/Ministry of Health.

## Results

For the simulators evaluation through the pilot study, 12 gynecology and obstetrics resident doctors, 15 undergraduate Medical students and 11 nurse midwives who participated in an obstetric emergency training using the simulators were invited to participate. We obtained 31 replies to the questionnaire: 10 resident doctors, 12 undergraduate medical students, and 9 nurse midwives, with an 81.58% response rate, similar in all groups.

The mean age of all respondents was 29.68 years (SD 6.36). Among midwives (already graduated and with specialization degree) the mean age was 35.44 (SD 7.45) years. Among residents, the mean age was 28.7 (SD 3.56) years and among medical students 26.17 (SD 4.24) years. All the medical students were in their fifth year of the Medical School and participated in the simulation less than 6 months ago. Among residents, two were in the first year, three in the second year and four in the third year of training. First-year residents participated in the simulation less than 6 months ago. Those from the second and third years have been participating in the simulations since the beginning of their residency, in a repeated and systematic way. MNM have, on average, 8.89 (SD 8.65) years of experience and all participated in the same emergency course with the use of simulators in the 24 months prior to the questionnaire application.

The questionnaire responses are summarized in Table 1.

**Table 1:** Perception of apprentices on the teaching with low-cost handmade simulators (n=31), Florianópolis, SC, Brazil, 2019

	I agree (totally or partially)	I neither agree nor disagree	I disagree (totally or partially)
I was satisfied with the use of the simulator	31	0	0
The very simple simulator(s) does(do) not allow for optimal learning	3	3	25
The very simple simulator(s) prevents the student from feeling in a real clinical setting	3	5	23
The class in which the simulator was used increased my theoretical knowledge	31	0	0
The class in which the simulator was used increased my ability to solve clinical problems	31	0	0
The class in which the simulator was used decreased my anxiety/stress to deal with situations like the one in the simulation	29	0	2
I was able to solve a clinical problem/situation after taking the course that used the simulator	19	5	7

The open-ended question about the use of the acquired knowledge in real situations was answered by midwives and medical residents. According to the answers, when simulated situations occur in the clinical practice, *the training helps the professional to feel safer and more confident, reducing anxiety and improving performance.*

*After the class with the simulator, I had to manage a post-partum hemorrhage case and could confidently follow the steps* (Resident of the 2nd year); *I alone solved a hemorrhage case* (Resident of the 3rd year); *Before the class with the simulator, I had too many difficulties even to identify the planes. After the class I feel safer and more confident. The residents, after training, have already taught some doctors in the service the new techniques of suturing* (Resident of the 2nd year); *I was faced with a shoulder dystocia case and felt calmer to resolve* (Resident of the 3rd year); *I acquired greater agility to resolve bleeding cases* (nurse midwife).

In the open space for observations, at least three respondents indicated that they have used or intend to use low-cost handmade simulators for the training of other professionals or in groups of pregnant women. This finding indicates that one of the innovations that the models bring, besides the security and confidence with the realistic simulations, is the possibility of an easy reproducibility in their contexts, making the knowledge more democratic.

## Discussion

We aimed to describe the developing process of low-cost handmade simulators that allow apprentices to acquire knowledge, increase skills and train attitudes regarding various obstetric procedures. An evaluation of the perceptions of students and professionals who used the simulators in a real-world training context was also carried out, adopting an evaluation methodology for simulators that allowed to address the reaction of the learners. In our sample, all those who answered the questionnaire were satisfied with the simulation. Apprentice satisfaction is often high in simulation environments^(^ 8 ^,^ 34 ^)^, the same happens for low-cost/artisanal simulators^(^ 17 ^,^ 19 ^)^.

The self-perception of the learning acquired was positive, with all respondents considering that the simulator class increased their theoretical knowledge and skills. A fact also observed in several studies, especially when the evaluation is performed by the apprentice themselves^(^ 18 ^-^ 20 ^,^ 34 ^)^. The use of simulators presents several advantages such as the possibility of repeating the procedure, correcting errors, and perceiving the difficulties (personal and inherent to the procedure)^(^ 35 ^)^. This is expected to improve performance in real-world situations.

Currently, the evidence is quite consistent to state that simulation-based training in Obstetrics improves knowledge and skills. The improvement of clinical and surgical practices is emerging with great consistency. Improvements in populational outcomes are less consistent, but there are some evidence mostly those related to neonatal outcomes^(^ 1 ^,^ 8 ^,^ 11 ^,^ 20 ^)^. The differences between low- and high-cost/high fidelity simulators are not established and warrants further studies^(^ 17 ^-^ 18 ^,^ 28 ^)^.

The evaluation of the hands-on application of the knowledge and/or skills acquired in the training - behavior and results in the Kirkpatrick classification - was done by the last three questions of the closed-ended questionnaire and the open-ended question. Among the respondents who are currently working in labor and delivery rooms and maternity services (residents and nurse midwives), most reported that the use of simulators helped them solve problems. The most cited were cases of postpartum hemorrhage, shoulder dystocia and perineal repair. Almost all respondents also believe that the course where the simulator was used reduced their anxiety/stress to deal with a situation like the one presented. A fact that is corroborated in other studies^(^ 17 ^,^ 19 ^,^ 36 ^)^.

As a pilot study, the study scope was restricted to the opinion and experience of the apprentices. Some apprentices considered that, because the simulators were simple, they did not allow the ideal learning. In fact, to allow the student to get involved with the simulation, it must be challenging and require effort to resolve. One of the questions was whether the simulator allowed the student to feel in a real clinical setting. Eight respondents agreed with this statement. The data presented here may indicates that the more advanced the learner, the greater the fidelity of the simulator needed for them to feel immersed in the simulation^(^ 36 ^)^.

One of the limitations of the study is the small number of respondents due to its pilot study nature. Additionally, there may be a courtesy bias in the responses received, although secrecy and confidentiality of the information source were guaranteed. We cannot rule out the hypothesis that precisely those less satisfied with the classes and simulations did not answer the questionnaire. Also because it is a pilot study, an objective assessment of knowledge before and after the simulation was not made, as would be ideal^(^ 8 ^)^. Furthermore, the design and nature of the study did not allow a complete assessment of the 4^th^ level of Kirkpatrick^(^ 8 ^,^ 20 ^)^, which is the impact on results, which would need a more comprehensive analysis.

The proposal of the project of handmade simulators is that people can access and reproduce the simulators, expanding the resources that teachers who work in the training of new professionals have at hand to increase the effectiveness of their educational strategies. The aim is to freely publish how to make the simulators and keep them as an open technology (*open source*). The publication of new ideas and new simulators and the reproduction and modification of existing ones is allowed and encouraged.

An unexpected finding of the original project and this study was the involvement of apprentices with the project and handmaking of the simulators, giving suggestions and actually participating in their development. This involvement favors the deepening of knowledge, since, to assemble a simulator, it is necessary to access and put into use knowledge of anatomy, obstetrics, surgical technique, physiology, etc. Additionally, the participation of the apprentices in the development process has created opportunities to learn knowledge, skills, and attitudes usually considered extracurricular, but useful and interesting for a richer and broader professional performance, such as research and search for materials, sewing, bricolage in general, creativity in problem-solving, adaptation to low-resource situations, etc. Besides, at least three people who answered the questionnaires mentioned that they have already developed or are planning to develop similar simulators for training, based on the use of those described here. No similar data were found in the literature, so this is an innovation of the work presented.

Further research can expand the knowledge about the development and use of simulators for practical training in Obstetrics, Nursing and other health areas. Specifically, evaluation studies with a larger number of participants, employing other methodologies that allow assessing not only the learners’ perception but also the effect on their practices and the concrete results of the training to reduce obstetric and neonatal complications.

This study demonstrated the effectiveness of handmade simulators, built by the teachers themselves and at a reduced cost to enhance Obstetrics teaching. It shows that it is possible not only that the simulators can be low-cost, but that they can be created by teachers and students, with good results. The use of this type of simulators went beyond the improvement in clinical practice, stimulating students to deepen their knowledge and even to develop new simulation environments. It is a pioneer study in Brazil and it is expected that the models tested will be replicated and used in other locations and situations. The study is also expected to stimulate further research in the area.

## Conclusion

The pilot study revealed that the apprentices perceive that simulators favor the expansion of theoretical knowledge and skills to solve clinical problems, in addition to the reduction of anxiety to deal with situations similar to those simulated. Simulation-based learning is widely recognized in the literature as an effective method in the context of health professionals’ training, and the availability of simple, low-cost simulators contributes to broadening access to this resource for students, teachers, and professionals. Open source technology allows and encourages these simulators to be reproduced and improved in other scenarios.
